# Supplementary material for: The causal relationship between the human gut microbiota and pyogenic arthritis: a Mendelian randomization study
Source: Front Cell Infect Microbiol. 2024 Nov 26;14:1452480. doi: 10.3389/fcimb.2024.1452480 (PMC11629706; doi:10.3389/fcimb.2024.1452480)
Supplement: Supplementary file 1 [file DataSheet1.zip › mendelian test/ebi-a-GCST90027613.csv_forest.pdf]

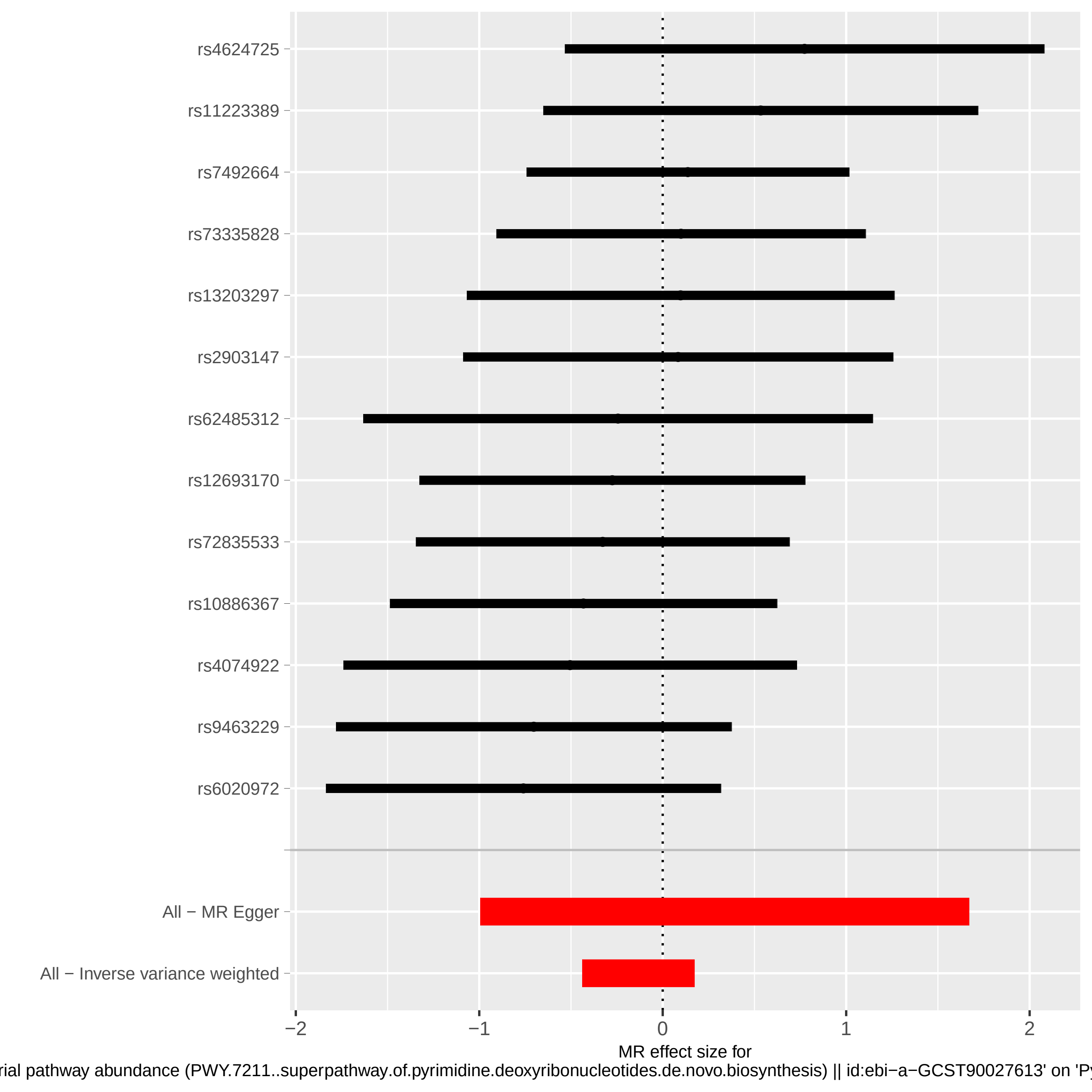
rs4624725
rs11223389
rs7492664
rs73335828
rs13203297
rs2903147
rs62485312
rs12693170
rs72835533
rs10886367
rs4074922
rs9463229
rs6020972
All − MR Egger
All − Inverse variance weighted
−2
−1
0
1
2
MR effect size for
rial pathway abundance (PWY.7211..superpathway.of.pyrimidine.deoxyribonucleotides.de.novo.biosynthesis) || id:ebi−a−GCST90027613' on 'Py
